# Management of Acquired Hemophilia A in Elderly Patients

**DOI:** 10.1155/2018/6757345

**Published:** 2018-11-13

**Authors:** Tomoya Yamaguchi, Naoko Kudo, Susumu Endo, Takeo Usui, Shinsaku Imashuku

**Affiliations:** ^1^Department of Internal Medicine, Uji-Tokushukai Medical Center, Uji 611-0042, Japan; ^2^Division of Hematology, Takasago-Seibu Hospital, Takasago 676-0812, Japan; ^3^Department of Laboratory Medicine, Uji-Tokushukai Medical Center, Uji 611-0042, Japan

## Abstract

This report describes six elderly patients with acquired hemophilia A (AHA), including four individuals aged ≥90 years. Bleeding symptoms were subcutaneous or intramuscular hemorrhage (*n*=4), hematuria (*n*=1), and hemorrhagic shock after tooth extraction (*n*=1). Factor VIII (FVIII) activity ranged from <1.0% to 3.0%, and anti-FVIII inhibitor titers ranged from 8.8 to 240 BU/mL. Treatment was administered at the discretion of the responsible physician. Hemostatic agents applied in the six patients comprised rFVIIa (NovoSeven®) (*n*=4), APCC (Feiba®) (*n*=2), and fresh frozen plasma/plasma exchange (*n*=1). Agents employed for inhibitor eradication comprised prednisolone only (*n*=3), prednisolone with cyclophosphamide (*n*=1), prednisolone with cyclosporine (*n*=1), and prednisolone with rituximab (*n*=1). In five patients, management was successful, with complete response. Treatment failed in the patient with the highest inhibitor level (240 BU/mL) in whom treatment with APCC (Feiba®; 100 U/kg/dose, three doses) and prednisolone (0.5 mg/kg/day) was followed by several episodes of relapse. The present data demonstrate that AHA severity shows wide variation in elderly subjects, indicating the necessity of individualized management.

## 1. Introduction

Acquired hemophilia A (AHA) is a potentially life-threatening bleeding disorder caused by antibodies against factor VIII (FVIII) [1]. Previous research found that, among patients who were assigned an appropriate diagnosis and treatment, mortality rates ranged from 6% to 8% [2]. AHA tends to occur in elderly patients with comorbidities [3], and patients with life-threatening bleeding secondary to AHA may be managed initially by nonhematologists at the emergency department [2]. The incidence of AHA increases with age, with 80% of the affected population encompassing individuals aged 65 years or older [4]. Treatment goals for AHA comprise hemostasis and inhibitor eradication [1, 3]. To control bleeding, previous authors have advocated the use of bypassing agents, including recombinant activated factor VII (rFVIIa; NovoSeven®) and/or activated prothrombin complex concentrate (APCC; Feiba®), as well as recombinant porcine FVIII [5]. Challenges in the management of AHA in elderly patients include (1) delayed diagnosis probably due to the relatively low prevalence of AHA, differentiation from other bleeding disorders, and the fact that patients typically present to nonhematologists; (2) the presence of comorbid disease often complicates the administration of prednisolone as well as the selection of hemostatic and immunosuppressive agents; (3) frequent need of anticoagulants in association with poor venous access; (4) high costs of therapy; and (5) thromboembolic risk when FVIII returns to normal. The present report describes the case histories of six elderly patients from Japan who were diagnosed with AHA. The report discusses issues that must be considered during the management of elderly AHA patients.

## 2. Case Reports

The summarized case histories of the six patients are presented below, who were treated at two institutions where board certified hematologists were available (Table 1). All cases were initially taken care at the emergency department except for one case. Cases 5 and 6 were described in previous reports [6, 7]. As treatment results, cases not attained partial remission (PR) defined as controlled bleeding with an FVIII activity level >50% on immunosuppression were considered no response (NR). Complete remission (CR) was defined as no bleeding with an FVIII activity level >50% with no detectable inhibitor (inhibitor <0.5 BU/mL) and without immunosuppression. In our study, assays on FVIII activity and inhibitor were performed at the SRL, Inc., where FVIII activity was measured by one-stage clotting and inhibitor by the Bethesda method with heat treatment.

### 2.1. Case 1

A 91-year-old female presented with a 4-day history of subcutaneous as well as intramuscular hemorrhages of the upper and lower extremities. In her past medical history, she had thoracic and lumber compression fractures. Laboratory investigations revealed a severe anemia (Hb 4.6 g/dL) with a prolonged APTT (93.3 sec; control 27.8 sec). Initially, she was taken care of with red blood cell (RBC; total 12 units) and fresh frozen plasma (FFP; total 8 units) transfusions. On day 5, interventional radiology was carried out to examine femoral vasculatures because of severe extravasation in the right thigh; however, no abnormalities were noted. On day 10 of admission, she was found to have an FVIII activity level <1.0% and an anti-FVIII inhibitor level of 110.6 BU/mL, in addition to positive lupus anticoagulant (LAC) (phospholipid neutralization method 14.5 sec; reference <7.9 sec). A diagnosis of AHA was therefore assigned with positive LAC. Following consultation with a hematologist, the patient received 36 doses (4.5 doses/day) of rFVIIa (NovoSeven®; 90 *µ*g/kg/dose) and prednisolone (1.2 mg/kg/day and then tapered). On day 33, combined cyclophosphamide (CPA; 1.7 kg/kg/day) and prednisolone therapy was initiated, which was administered for 5 weeks. Within 7 weeks, a CR was obtained.

### 2.2. Case 2

A 93-year-old male patient presented with hematuria, epistaxis, and a subcutaneous hemorrhage in the region of the right shoulder two weeks after admission for influenza B pneumonia. Laboratory investigations revealed anemia (Hb 7.5 g/dL), a prolonged APTT (73.1 sec; control 27.6 sec), and microscopic hematuria (>100/HPF). Five days later, the patient was diagnosed as having AHA when laboratory investigations revealed an FVIII activity level <1.0% and an anti-FVIII inhibitor level of 8.8 BU/mL. After consultation with a hematologist, the patient was administered six doses of rFVIIa (NovoSeven®; 90 *µ*g/kg/dose) over 2 days in combination with prednisolone (0.6 mg/kg/day and then tapered). Within 5 days, the hematuria and subcutaneous hemorrhages improved. AHA was controlled within 2 weeks, and a CR was obtained.

### 2.3. Case 3

A 92-year-old female presented with generalized subcutaneous hemorrhages for a few days. Three weeks before, she had undergone surgery for a left femoral trochanteric fracture. Because of anemia (Hb 7.3 g/dL) and a prolonged APTT (55.2 sec; control 29.0 sec), she was administered RBC (10 units) and FFP (2 units) transfusions, and oral prednisolone (0.5 mg/kg/day and then tapered) was started. On day 7 of admission, laboratory investigations revealed an FVIII activity level of 3.0% and an anti-FVIII inhibitor level of 10.0 BU/mL. A diagnosis of AHA was therefore assigned. The patient received two doses of rFVII (NovoSeven®; 90 *µ*g/kg/dose), and prednisolone (0.5 mg/kg/day and then tapered) was continued as maintenance therapy. AHA was controlled within 7 weeks, and a CR was obtained. The patient remained stable for 2 years. However, she then developed further subcutaneous hemorrhages. Laboratory investigations revealed an FVIII activity level of 6.2% and an anti-FVIII inhibitor level of 2.1 BU/mL. With a diagnosis of AHA relapse, reintroduction of prednisone (0.6  mg/kg/day) was performed.

### 2.4. Case 4

A 90-year-old male presented with a left gluteal intramuscular hemorrhage. The patient was in a state of chronic heart failure and was anemic (Hb 6.4 g/dL) with a prolonged APTT (69.2 sec; control 28.0 sec). Based on the information that he had previously been diagnosed as having AHA, a relapse of AHA was suspected, and the patient was immediately managed with RBC infusion (2 units), APCC (Feiba®; 100 U/kg/dose, three doses), and prednisolone (0.6 mg/kg/day and then tapered). Later, it was confirmed that the patient had an FVIII activity level <1.0% and an anti-FVIII inhibitor level of 240 BU/mL. With the above measures, the patient remained in a state of no bleeding but FVIII activity <50% over the subsequent several months. Thereafter, he developed repeat intermittent cutaneous hemorrhages, which required three further periods of hospitalization. During each admission, minimal doses of rFVIIa (NovoSeven®; 90 *µ*g/kg/dose, two doses each) were administered to control the bleeding with a limited effect. Due to the persistence of high anti-FVIII inhibitor levels, the patient was transferred to a tertiary center for further management.

### 2.5. Case 5

An 80-year-old male presented with soft tissue hemorrhages in the left forearm and right lower extremity for 3 weeks, which progressed compartment-like symptoms [6]. He was anemic (Hb 8.0 g/dL) with a prolonged APTT (78.4 sec; control 25.3 sec). In his medical history, the patient had been noted to have leukocytosis two years earlier and was diagnosed to have chronic neutrophilic leukemia (CNL) after admission. Laboratory investigations revealed an FVIII activity level <1.0% and an anti-FVIII inhibitor level of 190 BU/mL. A diagnosis of AHA was therefore assigned in association with CNL. The patient was administered 16 doses of APCC (Feiba®; 100 U/kg/dose) and four doses of rFVIIa (NovoSeven®; 90 *µ*g/kg/dose), and hemostasis was achieved. Inhibitor eradication was achieved using prednisolone (0.6 mg/kg/day and then tapered) and two doses of rituximab (375 mg/m^2^/dose). During the treatment for AHA, reactivation was noted around the 7^th^ week of treatment, but administration of two more doses of rituximab was successful in eradicating the inhibitor. Together with the treatment of AHA, the underlying CNL was also treated with hydroxycarbamide (Hydrea®; 500 mg/day) and controlled well. A CR of AHA was attained within 26 weeks.

### 2.6. Case 6

A 62-year-old female presented with hemorrhagic shock a few hours after a tooth extraction and was referred to the emergency department [7]. The patient had been treated for noncompensated liver cirrhosis during the past several years. Laboratory investigations revealed severe anemia (Hb 4.6 g/dL) with a prolonged APTT (>100 sec; control 28.1 sec) and low fibrinogen (58 mg/dL; reference 200–400). She was taken care of by RBC (4 units) and fresh frozen plasma (FFP; total 32 units) transfusions with hemodynamic and respiratory care until day 8 when laboratory investigations revealed an FVIII activity level <1.0% and an anti-FVIII inhibitor level of 11.9 BU/mL. A diagnosis of AHA was therefore assigned. However, due to the liver cirrhosis, hemostasis was attempted using plasma exchanges (two courses of 30 units each) rather than the administration of APCC (Feiba®) or rFVIIa (NovoSeven®). Inhibitor eradication was achieved using a combination of prednisolone (1.0 mg/kg/day and then tapered) and cyclosporine A (CSA; 3.0 mg/kg/day). AHA resolved within 10 weeks, and a CR was obtained. Unfortunately, the patient died of liver failure a year after the onset of AHA.

## 3. Discussion

In this report, four of the present patients were ≥90 years of age. All cases except for one were admitted through the emergency department with bleeding episodes, and one had undergone a recent surgical procedure (tooth extraction). Hemorrhages ranged from subcutaneous, soft tissue, and intramuscular bleeding episodes to epistaxis and hematuria. Severe hematuria in AHA has been described by Shander et al. [2]. AHA is classified as severe (FVIII activity <1.0%), moderate (1.0–5.0%), or mild (5.0–50.0%), while serum anti-FVIII inhibitor levels in AHA are generally classified as low or high. Previous authors proposed the following anti-FVIII inhibitor cutoff values: (1) low <5 BU/mL and high >5 BU/mL [8]; (2) low <10 BU/mL and high >10 BU/mL [9]; or (3) low <20 BU/mL and high >20 BU/mL [10]. With the exception of Case 3, all of the present patients had severe AHA. According to the criteria of Vautier et al. [10], three of the patients showed high inhibition (Cases 1, 4, and 5; 110.6, 190, and 240 BU/mL, respectively), and three showed low inhibition (Cases 2, 3, and 6; 8.8, 10.0, and 11.6 BU/mL, respectively). Tiede et al. [11] could identify prognostic factors (FVIII <1%, inhibitor >20 BU/ml, poor performance state, and malignancy) as predicting a poor outcome. In our series, three (Cases 1, 4, and 5) belonged to the poor outcome group.

Review of the present cases was performed retrospectively, and treatment of each case was determined at the discretion of the responsible physician rather than in accordance with a specific AHA protocol. The management of hemostasis and inhibitor eradication therefore differed on a case-by-case basis and was based on FVIII activity, anti-FVIII inhibitor levels, and comorbidities. Of the three patients with high inhibition, two required high doses of rFVIIa (NovoSeven®) with or without APCC (Feiba®) and were appropriately managed. In the remaining patient (Case 4), only three doses of APCC (Feiba®) were administered initially, but complete bleeding control was not achieved. Among the three low inhibitor cases, two attained a control with 2–6 doses of rFVIIa (NovoSeven®), while the patient with liver cirrhosis was successfully managed with FFP, plasma exchange, and prednisolone. To determine the efficacy of prednisolone in the hemostatic management of AHA, Vautier et al. [10] evaluated AHA based on FVIII activity (≥ or <1.0%) and inhibitor levels (≤ or >20 BU/mL) in a cohort of 24 AHA patients in France. The authors concluded that AHA patients with FVIII levels ≥1 % alone or in combination with an inhibitor level of ≤20  BU/mL could be treated using prednisolone only. Based on their criteria, Case 3 in the present series could have been treated with prednisolone alone. Instead, she was treated with two doses of rFVIIa (NovoSeven®).

Although individualized, treatment in the present series did not deviate substantially from that proposed in the AHA Guidelines of the Japanese Society on Thrombosis and Hemostasis [12]. These guidelines recommend the administration of (1) rFVIIa (NovoSeven®), APCC (Feiba®), or FX/FIIa (Byclot®) for hemostasis, and (2) prednisolone alone, or a combination of prednisolone (initial dose 1 mg/kg/day) and CPA (1-2 mg/kg/day), for immunosuppression. Although rituximab or CSA can be employed as a second-line therapy, the cost of these therapies is not reimbursed by the Japanese health insurance system. A key issue in terms of their use in hemostasis is that these drugs are expensive. For example, rFVIIa (NovoSeven®; 5 mg) costs ¥426,490 ($4,000) and is administered at 3 hr intervals, while APCC (Feiba®; 5,000 U) costs ¥933,000 ($8,800) and is administered at 8 hr intervals. If a patient weighing 50 kg is administered five doses per day of rFVIIa (NovoSeven®; 5 mg/dose) for 3 days, the total cost is ¥12,795,000 ($121,000). Similarly, when three doses per day of APCC (Feiba®; 5,000 U/dose) are administered for 3 days, the total cost is ¥8,400,000 ($79,000). Since the required dose of rFVIIa (NovoSeven®) or APCC (Feiba®) is dependent on hemorrhage severity and comorbidities, the doses and duration of treatment show wide variation. Previous authors reported median rFVIIa (NovoSeven®) doses of 38 over 3.9 days [13] and median APCC (Feiba®) doses of 7.5 over 4.0 days [14]. To limit medical costs and to obtain maximum effect, physicians' efforts are required.

In terms of inhibitor eradication, since usage of immunosuppressive agents for AHA may develop severe side effects (leukopenia, sepsis, prednisolone-induced diabetes, etc.), treatment depended on the inhibitor levels with use of low-dose prednisolone (maximum doses; 0.5–1.2 mg/kg/day). Among cases in the present high-inhibitor group, Case 1 was treated with prednisolone + CPA, while Case 5 (who had a comorbid disease of CNL) required both rituximab and prednisolone. AHA in Case 4 was refractory and persisted in a state of NR probably because he was treated with prednisolone only for inhibitor eradication. Expert review of the case suggests that more intensive inhibitor eradication was indicated. Cases in the low-inhibitor group were treated with prednisolone only (Cases 2 and 3) or prednisolone + CSA (Case 6). Prednisolone dosages and rate of tapering varied according to the degree of impaired glucose tolerance and other age-related factors. Given the potential adverse effects of high-dose prednisolone in elderly patients, rituximab appears to be an ideal agent for anti-FVIII inhibitor eradication in this population [15, 16]. However, use of rituximab for the treatment of AHA is not reimbursed by the Japanese health insurance system.

To facilitate the evaluation of treatment effect in AHA, Tiede et al. previously defined PR as the absence of active bleeding and an FVIII level >50 IU/dL in cases in which hemostatic treatment had been discontinued for >24 hr [11]. In their investigation of 102 AHA cases from Germany, PR was achieved in 83% of patients after a median of 31 days [11]. In our series, five of 6 cases attained an eventual CR although PR status with an interval ranged from 2 to 26 weeks. Expert review suggests that the case remained in NR was probably attributable to suboptimal clinical management. Finally, in the care of AHA in elderly patients, caution must be exercised for an increased thrombotic and cardiovascular risk that could be increased by the administration of hemostatic agents; however, no such events were noted in our patients.

In summary, elderly patients frequently present to emergency services with various bleeding episodes. Given the aging demographic in Japan, this is likely to increase. In view of its life-threatening potential, emergency department physicians must consider AHA when formulating a differential diagnosis in such cases. Also, since the severity of AHA is variable, individualized therapy is needed to provide the patients with the best care.

## Figures and Tables

**Table 1:** Clinical features of 6 elderly cases with AHA.

	Case 1	Case 2	Case 3	Case 4	Case 5	Case 6
Age/gender	91/F	93/M	92/F	90/M	80/M	62/F
Body weight (kg)	29.1	48.1	43.0	41.1	53.0	50.9
Symptoms	Subcutaneous, intramuscular hemorrhages at extremities	Soft tissue hemorrhages, hematuria	Generalized subcutaneous hemorrhages	Intragluteal hemorrhage	Soft tissue hemorrhages at forearm and legs	Hemorrhagic shock after tooth extraction
Comorbidities	LAC positive	After influenza B	After surgery	Heart failure	CNL	Liver cirrhosis
Hb (g/dL)	4.6	7.5	7.3	6.4	8.0	4.6
APTT (sec)	93.3	73.1	55.2	69.2	69.0	>100
FVIII activity (%)	<1.0	<1.0	3.0	<1.0	<1.0	<1.0
Anti-FVIII inhibitor (BU/mL)	110.6	8.8	10.0	240	190	11.9
Time to Dx and hemostasis (days)	10	5	7	0^†^	3	8
Agents for hemostasis (total doses)	rFVIIa (36)	rFVIIa (6)	rFVIIa (2)	aPCC (3)	aPCC/rFVIIa (16)/(4)	FFP/PE, PSL
Time from hemostasis to IS agents (days)	23	6	0	0	11	20
Agents for IS	PSL/CPA	PSL	PSL	PSL	PSL/Ritux	CSA/PSL
Time (weeks) to restoration of FVIII activity (>50%)	7	2	7	Not done	26	10
Undetectable inhibitor	7	2	7	Not done	26	7
Outcome						
CR/PR	CR	CR	CR	NR	CR	CR
Follow-up (years)	<1	<1	>2	<1	>3	1^*∗∗∗*^
Relapse/reactivation	No	No	Yes^*∗*^	NA	Yes^*∗∗*^	No

Abbreviations: Dx = diagnosis; CNL = chronic neutrophilic leukemia; LAC = lupus anticoagulant; aPCC = activated prothrombin complex concentrate; rFVII = recombinant factor VII; IS = immunosuppression; PSL = prednisolone; CPA = cyclophosphamide; Ritux = rituximab; CSA = cyclosporine A; FFP = fresh frozen plasma; PE = plasma exchange; CR = complete remission; NR = no remission; NA = not applicable; ^*∗*^relapse occurred 2 years after CR; ^*∗∗*^reactivation at the 7^th^ week of treatment; ^*∗∗∗*^died of cirrhosis-related hepatic failure; ^†^AHA previously diagnosed.
